# Reference standards for accurate validation and optimization of assays that determine integrated lentiviral vector copy number in transduced cells

**DOI:** 10.1038/s41598-020-79698-w

**Published:** 2021-01-11

**Authors:** Barbara S. Paugh, Lajos Baranyi, Andre Roy, Hua-Jun He, Lindsay Harris, Kenneth D. Cole, Moria Artlip, Caroline Raimund, Patricia S. Langan, Srikanta Jana, Rimas J. Orentas, Sheng Lin-Gibson, Winfried Krueger, Boro Dropulić

**Affiliations:** 1 Lentigen, a Miltenyi Biotec Company, Gaithersburg, MD USA; 2 National Institute of Standards and Technology, Gaithersburg, MD USA

**Keywords:** Immunotherapy, Preclinical research, Translational research

## Abstract

Lentiviral vectors (LV) have emerged as a robust technology for therapeutic gene delivery into human cells as advanced medicinal products. As these products are increasingly commercialized, there are concomitant demands for their characterization to ensure safety, efficacy and consistency. Standards are essential for accurately measuring parameters for such product characterization. A critical parameter is the vector copy number (VCN) which measures the genetic dose of a transgene present in gene-modified cells. Here we describe a set of clonal Jurkat cell lines with defined copy numbers of a reference lentiviral vector integrated into their genomes. Genomic DNA was characterized for copy number, genomic integrity and integration coordinates and showed uniform performance across independent quantitative PCR assays. Stability studies during continuous long-term culture demonstrated sustained renewability of the reference standard source material. DNA from the Jurkat VCN standards would be useful for control of quantitative PCR assays for VCN determination in LV gene-modified cellular products and clinical samples.

## Introduction

Gene therapy has emerged as an important paradigm for curative treatment of multiple indications in cancer immunotherapy, autoimmune and monogenetic hereditary diseases. In many cases lentiviral vectors (LV) have been the choice vehicle for gene delivery due to their robustness and broad applicability^1–11^. The dose of any medicinal product is a critical attribute when assessing its safety and efficacy. For gene modified cellular products, the dose is both the number of gene modified cells that are administered to the patient and the vector copy number of the transgene per cell, or VCN. Therefore, the VCN is a critical attribute when assessing the safety and efficacy of gene modified cell products. It is becoming increasingly imperative to establish reference materials (also commonly referred to as reference standards) that permit the accurate comparison of safety and efficacy of various lentiviral vector gene-modified cellular products, and for their monitoring in patients after their administration^12–14^.

Here we report the development of extensively characterized, renewable, VCN-defined cell line standards for use during establishment and validation of quantitation methods for determination of the number of integrated LV genomes in cell products and clinical samples. We found that we could accurately determine VCN using several orthogonal methods with concordant results obtained by independent laboratories and operators. We also found that the VCN was stable during continuous long-term culture of the source cells for the standards, indicating their suitability as a sustainable VCN reference standard. We show that for each of these cell lines a defined genomic integration site is present as measured by whole genome sequencing analysis, duplexed quantitative real time and digital droplet PCR. A genomic -identity assay was also developed and confirmed the GFP-expressing reference lentiviral vector used in this study. Finally, we show that expression of GFP driven by a constitutive promoter is consistent with the genomic copy number and can serve as an orthogonal method to confirm PCR based assays. We propose that the above reference standards would be useful for establishing accurate quantitative measures for dose of lentiviral vector gene-modified cellular products.

## Results

### Engineering of stable cell lines with defined copies of integrated lentiviral vector

Jurkat cells (Clone E6-1) were selected as the parental line to establish cell based lentiviral VCN reference standards. This immortalized T-cell line harbors a pseudodiploid genome, is easily maintained under standard suspension culture conditions and can be rapidly expanded to allow large scale preparation of genomic DNA. A third-generation lentiviral vector (LV) encoding green fluorescent protein (GFP) was selected for transduction of the cells at serial dilutions to limit the mean number of integration events to values ranging from one to five. The sequence identity in the lentiviral target region among existing vectors permits the generation of a reference standard with broad target range for both clinical and non-clinical applications.

**Figure 1:**
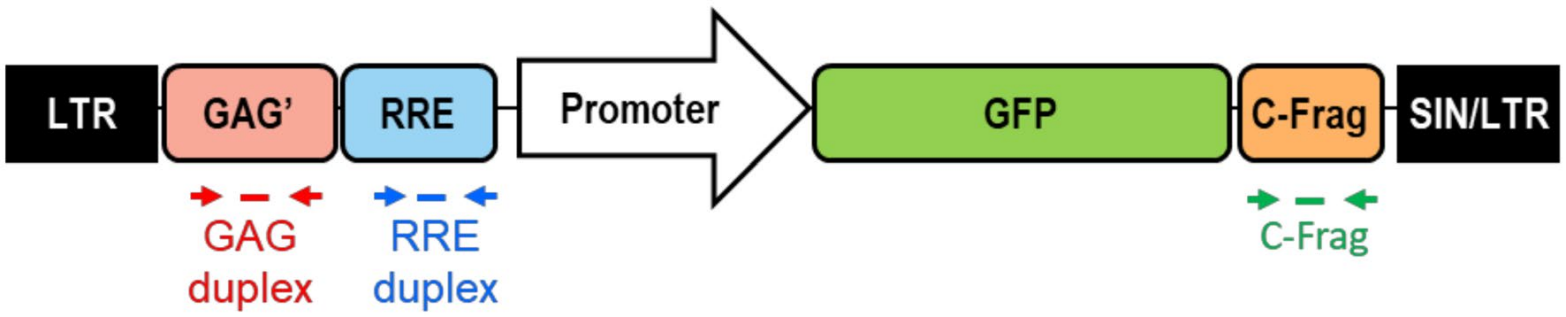
Schematic diagram of lentiviral vector used for development of Jurkat cell based VCN reference standards. Third generation lentiviral vector encoding green fluorescent protein (GFP) under the control of the human elongation factor 1 alpha (EF1α) promoter was used for stable transduction of Jurkat cells to engineer VCN reference standard cell lines. Selected single cell clones were characterized using three independent qPCR assays with primers and probe sets targeting three distinct sequences in the integrated lentiviral provirus DNA: GAG’, RRE and C-Frag Id tag. Additionally, RRE duplex primers and probes were used for ddPCR assay to evaluate performance of qPCR-based VCN assays and to measure vector copy number by an independent molecular test.

Lentiviral particles were manufactured at Lentigen by transient transfection of a third generation transfer construct and three packaging helper plasmids into HEK293T cells grown in suspension culture (Fig. 1). These particles were subsequently used to stably transduce Jurkat cells at defined multiplicity of infection (MOI = 0.2, 5 and 10). Following transduction, a dual series limiting dilution was performed to select candidate clones with the desired copy number of integrated LV. Clones containing one to four copies of integrated provirus were obtained, candidates selected based on growth pattern were expanded and genomic DNA was extracted for comprehensive characterization of the genomic DNA content in the candidate clonal cell lines.

### Concordant measurement of vector copy number in established clonal cell lines

**Figure 2:**
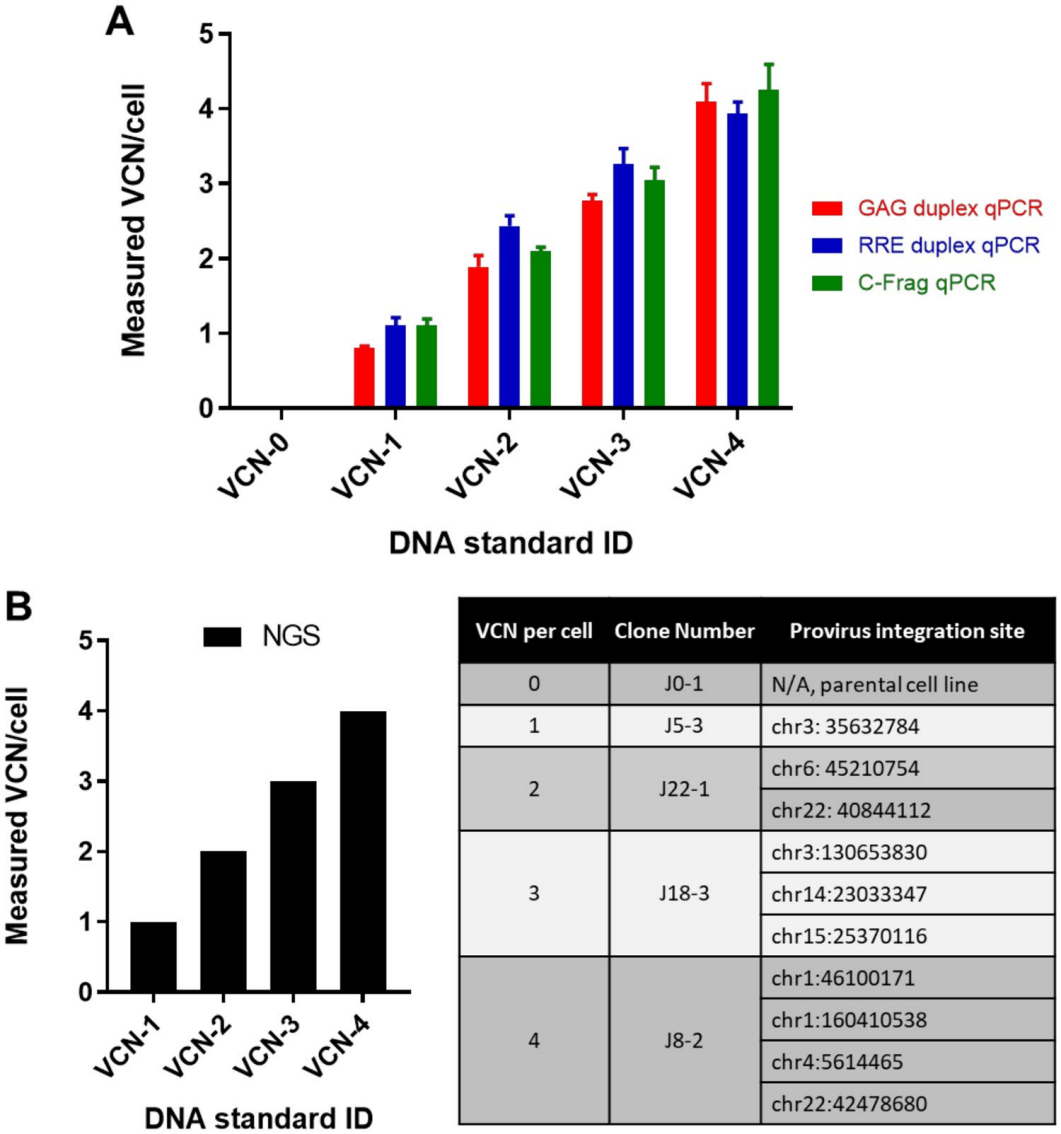
VCN reference standards characterization by qPCR and NGS. Genomic DNA extracted from clonally selected Jurkat cell lines was used to perform vector copy number analysis using three distinct qPCR assays and NGS. A) qPCR analysis was carried out using 200 ng of genomic DNA per reaction and three separate assays with primers/probe sets targeting three distinct sequences within the integrated vector sequence. GAG’ and RRE specific assays were designed and optimized as duplex assays with simultaneously run internal reference. C-frag qPCR was run as a single assay and then results were normalized to results from separately run qPCR for a reference gene. The graph represents a mean from at least two independent experiments where each reaction was run in triplicate. Error bars represent standard deviations for the means of the data sets. B) Next generation sequencing was performed on selected clones and confirmed presence of the entire proviral sequence integrated into the Jurkat cell genome which was consistent with expected VCN per cell for each clone. NGS mapped provirus integration sites and the identified insertion sites were validated using PCR and Sanger sequencing primers designed based on the flanking sequences of the mapped integration sites.

To screen and identify candidate clones, quantitative real-time PCR (qPCR) was performed with the extracted genomic DNA. Analysis of clones by qPCR entailed three separate non-redundant assays. Assay 1 used a primer set and probe specific for a synthetic ID sequence tag (C-frag) upstream of the 3′-sinLTR in the integrated lentiviral genome. This assay was normalized via qPCR for a target sequence in the single copy RPPH1 (Ribonuclease P RNA Component H) gene and the two reactions were performed separately using assay conditions optimized individually for each C-Frag and RPPH1 primer and probe set. Assay 2 used a primer set and probe specific for a selected sequence within the gag’ portion in the 5′ sequence of integrated lentiviral genome. This assay was normalized via qPCR for a target sequence in the single copy PTBP2 (Polypyrimidine Tract Binding Protein 2) gene and the two reactions were performed in a single tube assay using optimized conditions for both gag and PTBP2 primer and probe sets (duplexed assay)^15^. Assay 3 targeted the RRE (Rev response element) sequence located downstream of gag’ in the integrated lentiviral genome. This assay was developed and optimized as a duplex assay and the results were normalized via qPCR for a target sequence in the single copy RPL32 (Ribosomal Protein L32) gene^16^. All three qPCR assays yielded concordant results indicating that lentiviral target sequences were present in each evaluated clone after integration (Fig. 2A). Furthermore, since each qPCR assay utilized different reference gene target sequences with distinct chromosomal locations in the human genome (RPPH1 gene on chr14:20,343,071–20,343,411; PTBP2 gene on chr1:96,721,605–96,823,739 and RPL32 gene on chr3:12,834,485–12,841,582) the consistent results highlight the Jurkat cell line as a suitable choice for development of VCN reference standard.

**Figure 3:**
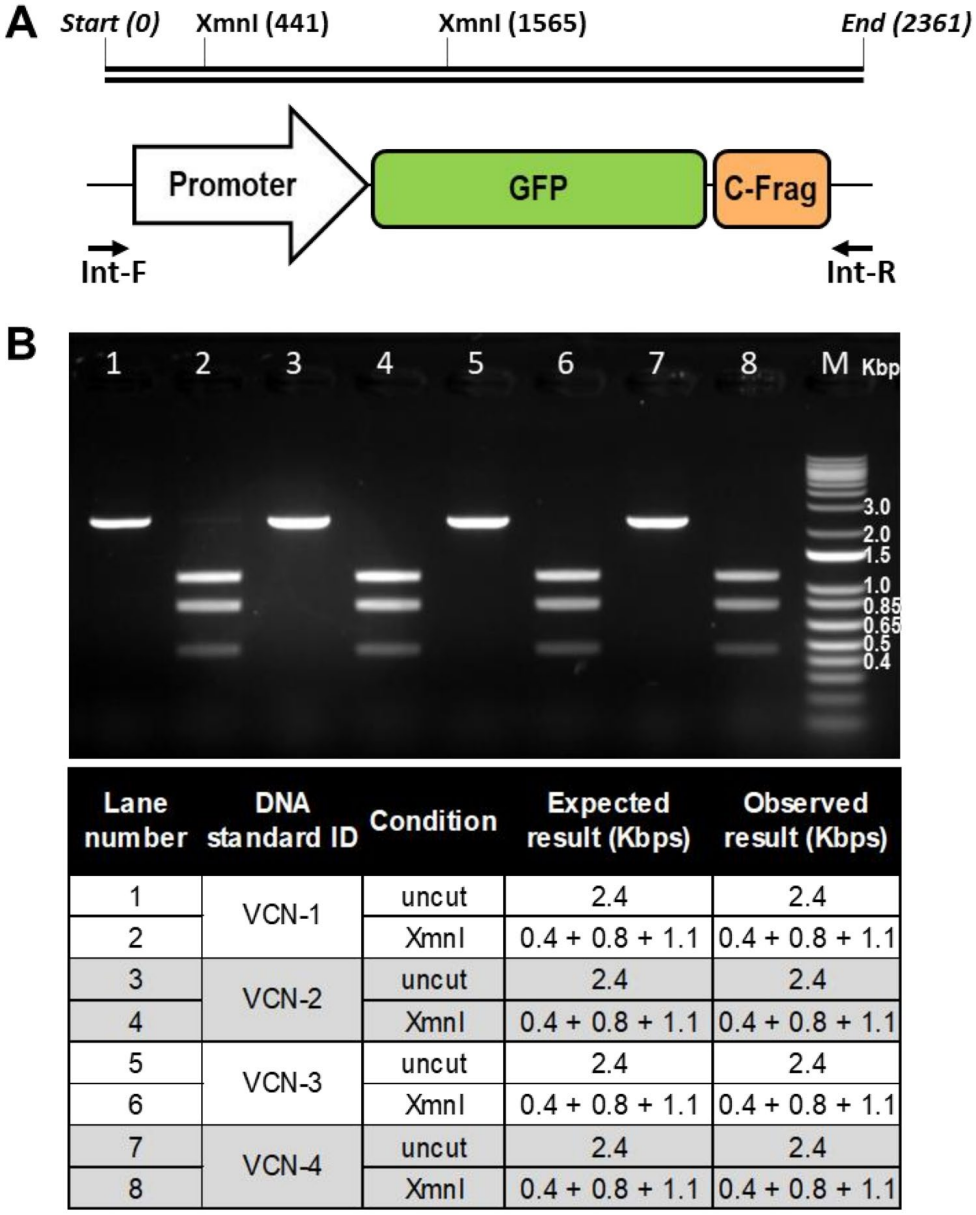
Genomic identity assay confirmed identity of inserted proviral sequence in established clonal cell lines. (**A**) The identity of the integrated proviral sequence was analyzed using PCR-based assay with Int-F and Int-R PCR primers flanking the sequence of a transgenic cassette including promoter sequence, open reading frame of GFP and C-Frag Id tag. (**B**) Genomic DNA extracted from each evaluated cell line was used as template material for PCR reaction and a single 2.4 Kbp amplicon was observed for each clone. Generated amplicons were purified and digested using XmnI restriction enzyme, which yields a unique three fragment pattern on the agarose gel.

To further characterize the engineered cell lines whole genome sequencing was performed on selected clones with one, two, three and four copies of the integrated vector. Next generation sequencing mapped integration sites for the provirus and showed VCN numbers concordant with quantitative real-time PCR based assays (Fig. 2B). Notably, observed integration sites were within intergenic or intronic regions of the genome and did not exhibit negative impact on stability of the engineered cell line or growth kinetics. Additionally, genomic identity of the integrated proviral sequence was evaluated using a standard PCR-based assay developed to amplify a fragment of the integrated vector DNA sequence including the promoter sequence, open reading frame of GFP and C-Frag Id tag. This PCR assay generated a single distinct amplicon of expected size and XmnI based restriction fragment length analysis resulted in a unique set of fragments of expected size confirming sequence identity for each of the established VCN standard cell lines (Fig. 3). Furthermore, Sanger sequencing was performed on generated PCR products and the obtained consensus sequences were identical to the reference sequence.

**Figure 4:**
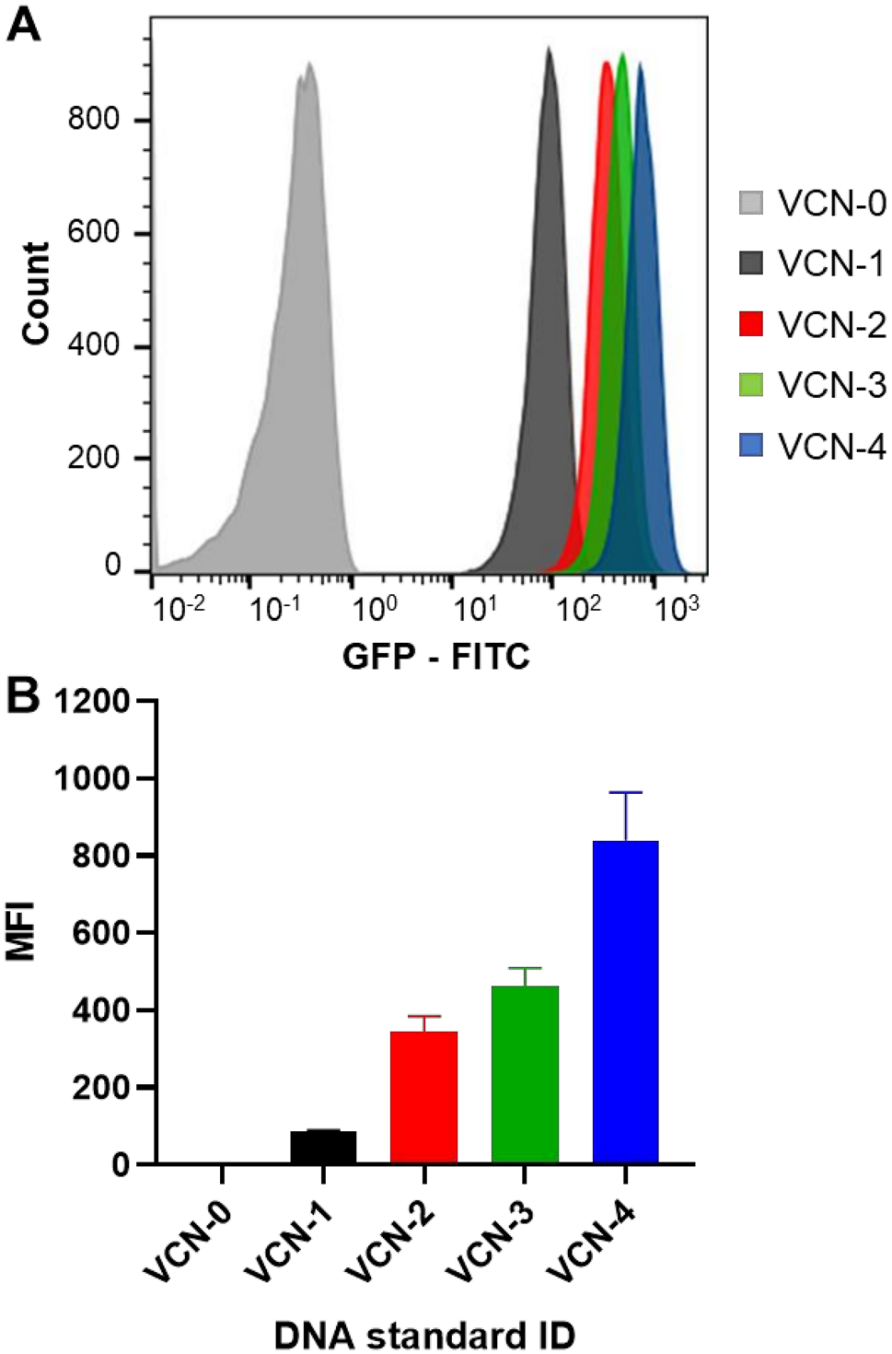
GFP mean fluorescence intensity expression correlates with vector copy number. Developed clonal Jurkat cell lines with a defined number of copies of integrated vector were evaluated by flow cytometry to measure expression of GFP, which is encoded by the integrated vector sequence under the control of a constitutive promoter. (**A**) Representative histogram plot showing increased expression of GFP observed in engineered Jurkat cell lines with higher VCN per cell. (**B**) GFP mean fluorescence intensity (MFI) from three independent measurements is consistent with the genomic vector copy number for each evaluated cell line. Error bars represent standard deviations for the means of the data sets.

The developed clonal Jurkat cell lines were also evaluated by flow cytometry for GFP expression encoded in the integrated vector sequence. Expression of GFP driven by a constitutive promoter is consistent with the genomic copy number and can serve as an orthogonal measure to PCR based assays (Fig. 4).

### Characterized cells lines stably retain integrated provirus

**Figure 5:**
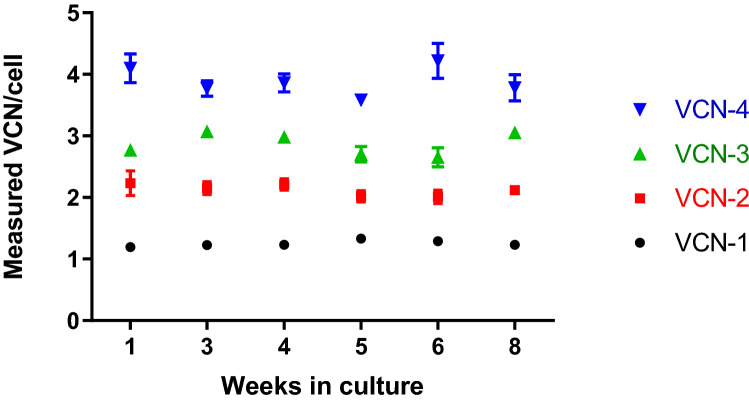
VCN reference standards stability in cell culture. Established VCN standard cell lines were cultured for 8 weeks using suspension cell culture conditions specific for Jurkat cells. Each week cell pellets were collected to evaluate VCN stability in culture. Genomic DNA was extracted from collected cell pellets and VCN per cell was measured using the GAG duplex qPCR assay. No VCN change was observed during 8 weeks propagation time in culture. Error bars represent standard deviations for the means of replicates.

A critical property for a VCN reference standard is its continued availability requiring sustained culture of the cell material without VCN change. To determine whether the engineered cell lines retain the integrated provirus, long-term cultures of the selected cell clones were performed over the course of eight weeks. At selected timepoints during this period, cells were harvested, and their genomic DNA was extracted for subsequent VCN interrogation by quantitative real-time PCR. No VCN changes were observed for all samples analyzed (Fig. 5) indicating stable maintenance of the integrated lentiviral vector copies and highlighting these cell lines as a stable renewable source for future VCN reference standard preparations.

### Robustness of VCN standards and assays in multiple laboratories

**Figure 6:**
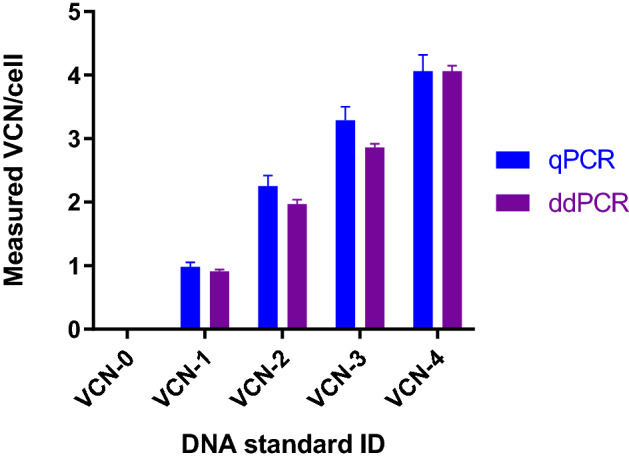
Established VCN genomic DNA standards showed uniform performance and accuracy when evaluated by independent laboratory. Genomic DNA extracted from developed VCN standard cell lines was evaluated by an independent laboratory using the RRE duplex assay for VCN determination by qPCR and ddPCR. The qPCR results represent a mean of four independent qPCR runs performed by two independent operators at NIST laboratory on two different qPCR instruments and using two different DNA inputs, with four replicates run for each condition (in total 32 qPCR data points for each tested VCN standard). The ddPCR results are concordant with the qPCR assay results and each data point represents a mean of two independent ddPCR runs performed on Bio-Rad QX200 instrument by two independent operators and using two different DNA inputs (in total 16 ddPCR data points for each tested VCN standard). The error bars are the sample standard deviations for the means of the data sets, with N = 32 for qPCR and N = 16 for the ddPCR results.

Uniform performance and accuracy among platforms, laboratories, operators and distinct assays is an essential attribute of a successful reference standard^14,17^. To assess robustness of Lentigen developed VCN reference standards and assays, extracted genomic DNA was evaluated by an independent laboratory at the National Institute of Standards and Technology (NIST, Gaithersburg, Maryland, USA). Primers and probes for RRE duplex assay were used for VCN determination by qPCR and ddPCR. The qPCR assays were carried out in four replicates for each condition by two independent operators using two different qPCR instruments, ABI 7500 and ABI ViiA 7 and two different DNA inputs (25 ng and 125 ng per reaction). A total of 32 qPCR data points were generated for each tested VCN standard and the reported mean of all measurements is concordant with the data reported by Lentigen (Fig. 6). Furthermore, the evaluation of genomic VCN reference standards also included VCN determination by ddPCR assay by two independent operators and using two different DNA inputs (10 ng and 20 ng per reaction). In total 16 ddPCR data points for each tested VCN reference standard were collected with results concordant with the qPCR results generated by NIST and Lentigen, highlighting consistency between different laboratories, operators and instruments (Fig. 6).

## Discussion

We report the development and characterization of a set of vector copy number (VCN) defined cell lines as a potential source of reference standards for assay validation. The cell lines allow for precise and robust determination of integrated lentiviral VCN in gene-modified cell products and clinical samples.

We selected a transfer vector with a lentiviral target sequence that is present in a broad range of first, second and third generation lentiviral constructs^17^. This allowed us to produce VCN defined reference standards for most vectors currently used in clinical and non-clinical settings.

We sought to obtain VCN numbers ranging from one to five integration events per cell since regulatory bodies currently limit the VCN to five in any clinical cell product. The clonal cell lines comprising this reference standard contain one copy increments up to four copies per cell as determined by PCR and NGS based methods. The genomic DNA from these cell lines with stable, defined DNA content yield uniform VCN results across orthogonal platforms, independent laboratories and operators. Thus, our reference DNA provides an accurate benchmark that can be used for validation of VCN quantitation protocols in a site independent manner and permits the robust and precise determination of VCN at any clinical site that manufactures and clinically applies LV gene-modified cellular products.

The major advantage of the stable cell lines with defined copies of integrated lentiviral vector is a consistent standard which is established from renewable source material. This material will provide greater consistency among assays as this DNA material is ready to use after purification which will reduce pipetting errors and decrease variability across sites, equipment and laboratories that could be introduced during the two-step process when preparing the standard by spiking human genomic DNA with plasmid DNA. Furthermore, transduced cell line-based standards provide material generated using a method that reflects the workflow required to test gene engineered cell populations, and this is closer to the real-life application of analyzing transduced cells. An additional advantage is cost, the transduced cell master banks serve as a permanent analytical library, without additional reagents required.

We chose Jurkat cells as a parental cell line since these cells were derived from immortalized T-cells and on the basis of their pseudodiploid genome that is maintained stably over many cell generations. A second criterium to select Jurkat cells, a T cell line, as the cell substrate of choice was due to the wide use of T-cells in adoptive cell cancer immunotherapy^18^. This choice proved to be prudent since we did not observe any VCN changes in the selected cell clones over a continuous eight-week culture period. Thus, the engineered cells provide a stable and renewable source of VCN reference standard that can be used for a variety of clinical and non-clinical applications. This set of VCN defined LV-transduced cell reference standards therefore provides a unique resource essential for establishing validated and standardized VCN assays for accurate quantitation of integrated LV genomes in cell products and clinical samples.

## Methods

### Establishment and propagation of engineered VCN standard cell lines

Jurkat cell line (Clone E6-1) was purchased from American Tissue Culture Collection (ATCC, Manassas, VA). Lentiviral VCN reference standard cell lines were generated by stable transduction of Jurkat cells with lentiviral vector encoding green fluorescent protein (Lentigen Technology, Inc., Gaithersburg, MD) at defined MOI (0.2; 5 and 10) and subsequent selection of single cell clones by dual series of limiting dilutions. Parental Jurkat cell line and generated VCN reference standard clonal Jurkat cell lines were cultured in RPMI-1640 growth medium (ATCC, Manassas, VA) supplemented with 10% heat-inactivated fetal bovine serum (FBS, Hyclone, Logan, UT) and 2 mM LGlutamax (Thermo Fisher Scientific, Grand Island, NY). Cultures were maintained at a cell concentration between 2 × 10^5^ and 1 × 10^6^ viable cells/mL by addition of fresh medium.

### Quantitative real time PCR (qPCR)

Integrated vector copy number (VCN) was measured using TaqMan technology (Applied Biosystems, Foster City, CA, USA). The minimum information for publication of quantitative real-time PCR experiments (MIQE) was followed during the assay development and for the measurements of the samples^19^. Genomic DNA was extracted from selected single cell clones from 1 × 10^6^ cells using DNeasy Blood and Tissue Kit from Qiagen and 200 ng of DNA was used per qPCR reaction. To generate a standard curve for absolute quantification of integrated vector copies, serial dilutions of plasmid standard were used (10^6^, 10^5^, 10^4^, 10^3^, 10^2^ and 10 copies/µL). Three independent qPCR assays were performed using custom primer–probe sets synthesized by Integrated DNA Technologies (IDT, Newark, NJ): 1) GAG-duplex assay using primers and probes targeting GAG sequence of the integrated vector and PTBP2 reference gene sequence in the host genome; 2) C-Frag single target assay using primers and probes binding to a unique ID tag sequence at the 3′ end of the Lentigen manufactured lentiviral vector 3) RRE-duplex assay using the following primers and probes targeting RRE sequence in the lentiviral vector and RPL32 reference gene in the host genome:

- RRE Forward Primer: 5′- AAACTCATTTGCACCACTGC -3′
- RRE Reverse Primer: 5′- AATTTCTCTGTCCCACTCCATC -3′
- RRE Probe: 5′FAM/- TGTGCCTTGGAATGCTAGTTGGAGT -/3′BHQ_1
- RPL32 Forward Primer: 5′- CAAGGAAAGACGAGCTGTAGG -3′
- RPL32 Reverse Primer: 5′- GGGCAGTTGCATCTTCATATTC -3′
- RPL32 Probe: 5′HEX/- AGCTGCAGGCAGAAATTCTGGTAGT -/3′BHQ_1

TaqMan Fast Advanced Master Mix (Life Technologies, Carlsbad, CA) was used and DNA was amplified using Bio-Rad CFX96 thermocycler and the following qPCR conditions: 2 min at 50 °C followed by initial denaturation step at 95 °C for 10 min, followed by 40 cycles of denaturation at 95 °C for 15 s and annealing/extension at 60 °C for 30 s. This qPCR conditions were further tested by independent laboratory using ABI 7500 and ABI ViiA 7 instruments and two different DNA inputs (25 and 125 ng per reaction). The VCN per cell was calculated using the following formula: VCN per cell = qPCR copy#/µL of LV vector genome target/qPCR copy#/µL of reference target × 2.

### Digital droplet PCR

Digital droplet PCR (ddPCR) was performed as secondary platform to quantify integrated vector copy number (VCN) using the same set of RRE/RPL32 primers and probes as for qPCR duplex assay and Bio-Rad ddPCR Supermix for probes (Bio-Rad, Hercules, CA).VCN was determined with the Bio-Rad QX200 droplet digital PCR system as per the manufacturer’s instructions. All the assays worked well according to the minimal information for publication of quantitative digital PCR experiments guidelines^20^. Two different inputs of genomic DNA (10 and 20 ng per reaction) were used to generate droplets with QX200 droplet generator. Using an Applied Biosystems Veriti 96-well thermal cycler (Life Technologies, Carlsbad, CA), droplets were amplified to end point by heating to 95 °C for 10 min followed by 40 cycles of 95 °C for 30 s and 62.2 °C for 60 s, with a final heating step of 98 °C for 10 min. The plate was then placed into the QX200 droplet reader and data were collected and analyzed using the manufacturer’s QuantaSoft software.

### Next generation sequencing

Whole genome sequencing analysis of lentiviral vector integration sites in Jurkat cell line genome was performed using Illumina TruSeq technology. Fragmented genomic DNA from selected clones was used to prepare NGS libraries with the NEXTflex Rapid Library Prep Kit and sequencing was performed on the Illumina HiSeq 4000 platform. At least 650 M PE150 reads per sample were generated. Raw reads were trimmed and aligned to the vector reference sequence. Reads spanning an integration site in which one end of the sequence stemming from the integrated vector and the other anchored in the human genome resulted in a partial alignment were obtained using bowtie in local alignment mode. These reads were sorted by chromosomal reference position and used to generate a filtered bam file. The soft-clipped reads were then converted into FASTA format for batch based pairwise mapping using the BLAT algorithm. Valid soft-clipped reads within vector sequence must align to position 0–400 and 3600–4000 of the vector. Paired reads not fitting the constraints were excluded and the next pair iteratively assessed. For each integration site, supporting soft-clipped reads must have included beginning and ending of the vector sequence. Such positions were considered as possible integration sites unless the sequencing depth was low and only one side of the vector integration site was covered. For each soft-clipped read, the attributes for determining the top BLAT hit were partial alignment and alignment breaking position consistent with the soft-clipped position. Identified insertion sites were validated using PCR and Sanger sequencing primers designed based on the flanking sequences of the mapped integration sites.

### Genomic identity assay

PCR-based identity assay was performed using genomic DNA extracted from selected clones and PCR primers targeting selected sequences of the integrated vector.

The following Int-F and Int-R PCR primers were designed to flank the sequence of a transgenic cassette including promoter sequence, open reading frame of GFP and C-Frag Id tag:

- Int-F Forward Primer: 5′-CAGTGCAGGGGAAAGAATAGTAGAC-3′
- Int-R Reverse Primer: 5′- GTGGCTAAGATCTACAGCTGCC-3′

Defined vector sequences were amplified using Q5 High-Fidelity DNA Polymerase mix (New England Biolabs, Ipswich, MA) and resulting PCR products were digested using the restriction enzyme XmnI for a unique three fragment pattern using agarose gel electrophoresis. The ethidium bromide stained gel was imaged using Odyssey Fc Imaging System and Image Studio Software for image acquisition using default settings. Additionally, sequence of amplicons was confirmed by Sanger sequencing.

### Flow cytometric analysis

GFP expression of clonal Jurkat cell lines was determined by flow cytometry. Data acquisition was performed using a MACSQuant10 Analyzer (Miltenyi Biotec, Bergisch Gladbach, Germany). To exclude non-viable cells, a gate was set on the live cell population using the SSC vs FSC plot. FLOWJO software 10.0.8 was used for data analysis. GFP mean fluorescence intensity (MFI) from three independent measurements was calculated. Statistical analysis was performed using GraphPad Prism Version 8.1.3.
